# The relationship between chronic type III acromioclavicular joint dislocation and cervical spine pain

**DOI:** 10.1186/1471-2474-10-157

**Published:** 2009-12-16

**Authors:** Stefano Gumina, Stefano Carbone, Valerio Arceri, Alessandro Rita, Anna R Vestri, Franco Postacchini

**Affiliations:** 1 Department of Orthopaedics and Traumatology, University of Rome "Sapienza". Piazzale Aldo Moro, 5. Rome, Italy; 2 Department of Experimental Medicine, University of Rome "Sapienza". Piazzale Aldo Moro, 5. Rome, Italy

## Abstract

**Background:**

This study was aimed at evaluating whether or not patients with chronic type III acromioclavicular dislocation develop cervical spine pain and degenerative changes more frequently than normal subjects.

**Methods:**

The cervical spine of 34 patients with chronic type III AC dislocation was radiographically evaluated. Osteophytosis presence was registered and the narrowing of the intervertebral disc and cervical lordosis were evaluated. Subjective cervical symptoms were investigated using the Northwick Park Neck Pain Questionnaire (NPQ). One-hundred healthy volunteers were recruited as a control group.

**Results:**

The rate and distribution of osteophytosis and narrowed intervertebral disc were similar in both of the groups. Patients with chronic AC dislocation had a lower value of cervical lordosis. NPQ score was 17.3% in patients with AC separation (100% = the worst result) and 2.2% in the control group (p < 0.05). An inverse significant nonparametric correlation was found between the NPQ value and the lordosis degree in the AC dislocation group (p = 0.001) wheras results were not correlated (p = 0.27) in the control group.

**Conclusions:**

Our study shows that chronic type III AC dislocation does not interfere with osteophytes formation or intervertebral disc narrowing, but that it may predispose cervical hypolordosis. The higher average NPQ values were observed in patients with chronic AC dislocation, especially in those that developed cervical hypolordosis.

## Background

Treatment of Rockwood type III acromioclavicular (AC) dislocation, which is characterized by a complete tear of the acromioclavicular and coracoclavicular ligaments with 25% to 100% upper dislocation of the clavicle, is still a motive for discussion. Literature reviews show that the rate of patients that obtained satisfactory results after conservative treatment is not much different from patients that underwent surgical procedures [1-4]. This conclusion becomes apparent when the shoulder function is globally considered. When the activities of daily living, ability to functionally use the arm, range of motion, pain, strength, are separately considered, outcomes may be different as they depend on age, gender, cultural level and job. Meta-analyses and retrospective studies show that the immobilization time is shorter and that going back to work and sport activities happens faster in conservatively treated patients. Surgically treated patients are however more satisfied in terms of their subjective shoulder pain, mobility and appearance [1]. Gstettner et al. [5] observed that calcifications of the coracoclavicular ligaments occurred more frequently in conservatively treated patients even though they don't seem to be cause of such symptoms. Controversies arise when discussion is focused on shoulder strength. Wojtys and Nelson [6] found that although their non surgical treated patients had the same amount of strength in their injured and uninjured shoulder, they experienced more discomfort with increased activity levels. At the follow up, Galpin et al. [7], found that despite different treatments, the range of motion and strength were similar between non- and operated groups. Tibone et al. [8] argued that shoulder strength is not significantly affected by conservative treatment; while Phillips et al. [1] reported that it is frequently normal or near normal in both groups. Finally, the infection rate is obviously higher in surgically treated patients.

To our knowledge, there are no study relating cervical spine symptoms to type III acromioclavicular separations. We propose a correlation between conservatively managed type III acromioclavicular dislocation and cervical spine symptoms because the clavicle and cervical spine are directly connected by means of the trapezius muscle. This hypothesis is also supported by Larsson et al. [9] who scrutinized the physiology of neck-shoulder pain and trapezius dysfunction based on the most recent scientific literature. Thus, we supposed that the upper migration of the lateral end of the clavicle may modify the biomechanics of this muscle, and we hypothesized that this condition may have repercussions on the cervical spine.

## Methods

The study group is represented by 34 consecutive patients who, with an average of 38.3 months earlier (range: 25- 49 months, S.D. 4.2 months), supported a Rockwood type III AC dislocation. The diagnosis was clinically and radiographically established (AP view, axillary and the stress view of the involved shoulder). All these patients were conservatively treated with a sling or a figure of eight splint for 30-40 days. The right side was affected in 22 cases. None of them had had radiculopathies of the upper limbs nor cervical symptoms (for more than 7 consecutive days) before the injury. None of the patients complained about cervical spine injury concomitant or before the shoulder trauma.

All patients were submitted to a radiographic examination of the cervical spine (AP and lateral view) in a standing position. Side roentgenograms of the cervical spine were taken during the neutral position. The patient stood at a distance of 120 cm from the tube with the shoulder in contact with the roentgenographic plate. Osteophytosis were determined to be positive when exceeding 2 mm [10,11]. These criteria have been regarded as the best available for epidemiologic researches and they have been used in large cross-sectional studies [12-15]. The degree of narrowing of the intervertebral disc was evaluated at each segment from the level of C2 to C7 according to the method suggested by Hayashi et al. [10] A disc was considered narrow when the maximal intervertebral disc space was less than 4 mm [10,11].

The magnitude of the cervical curve was measured on the lateral view radiograph from C2 to C7. The measurement was obtained by joining perpendiculars to lines drawn parallel to the inferior end plates of C2 and C7 [16]. We have considered as normal a mean Cobb's angle of 27°, that corresponds to the average value registered by Harrison et al. [17] in a group of normal, asymptomatic subjects.

One hundred healthy volunteers without shoulder pathologies and cervical pain symptoms (for more than 7 consecutive days) were recruited as a control group for this and other studies. Subjects were provided with information booklets explaining the aim of the study and informed consent documents were signed before their participation. They agreed to participate in the study, which was approved by the local Ethics Committee (Comitato Etico - Azienda Policlinico Umberto I°). Each radiograph was checked by one of the authors (S.G.).

**Figure 1:**
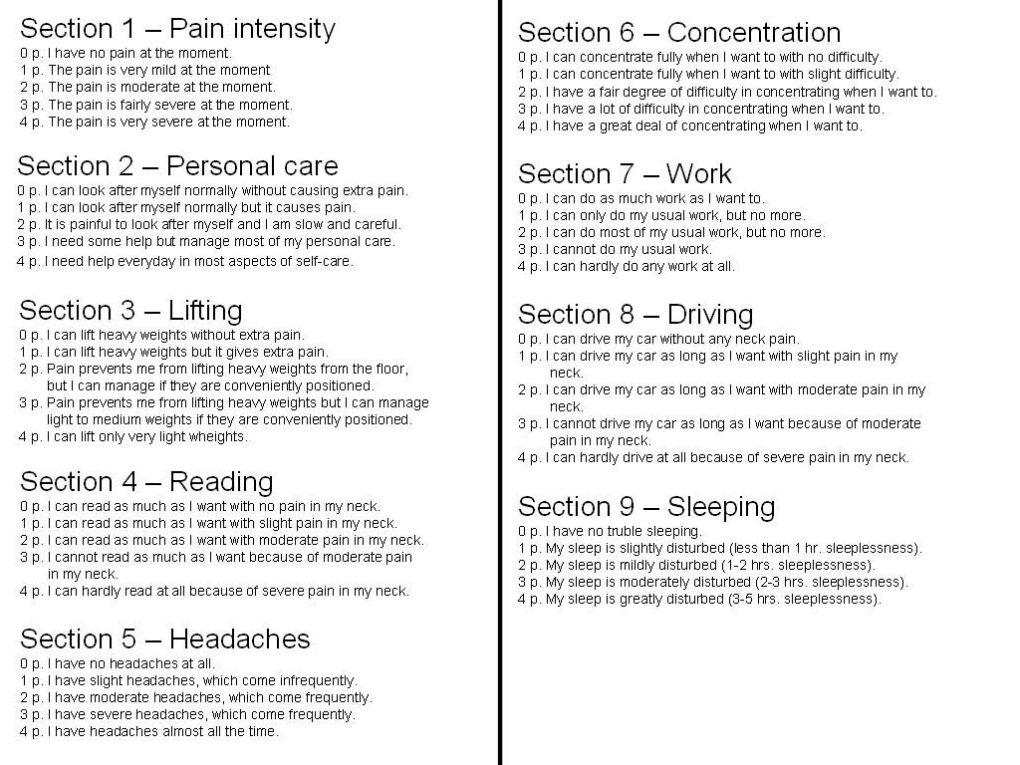
**The Northwick Neck Pain Questionnaire **[18].

To quantify subjective cervical symptoms, the Northwick Park Neck Pain Questionnaire (NPQ, Fig. 1) was administered [18]. The questionnaire checked the patient's symptoms and a score was obtained. The questions covered the many activities which are likely to cause neck pain. The 9 sections investigated are: pain intensity, sleeping, numbness, duration, carrying, reading-television, working/housework, social and driving. Each section is scored between 0-4 and summated. NPQ percentage score is calculated: total score/36 × 100%. The best result is 0%, the worst 100%.

For narrowing of intervertebral disc and osteophytosis, before and after dichotomisation based on the mean lordosis value, data were analysed with the Chi-Square test. Statistical analyses were performed using the Mann-Whitney Test for results of the Neck Pain Questionnaire and of the degree of lordosis. Nonparametric correlations between Neck Pain Questionnaire and degree of lordosis were calculated with the Spearman's Rho test. All statistic analyses were considered significant at the 0.05 level.

## Results

The average age was 46.8 and 45.8 years in the studied and the control group, respectively (p = 0.46). Out of studied group, 32 were males and 2 females, with an average age of 46.8 years (range, 24-69 years, S.D 9.3 years). Out the control group, 71 were males and 29 females with an average age of 45.4 years (range: 39-66, S.D. 9.3 years).

**Figure 2:**
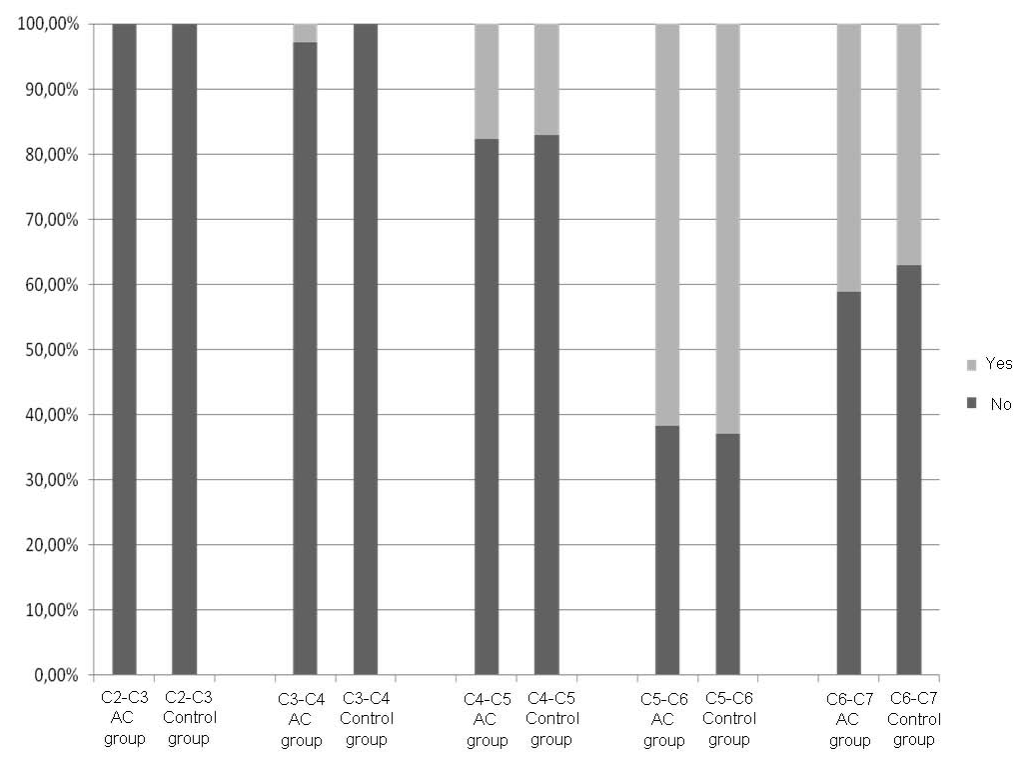
**Graphic representation of the prevalence of cervical spine osteophytes at each intervertebral level (p value from 0.08 to 0.93)**.

**Figure 3:**
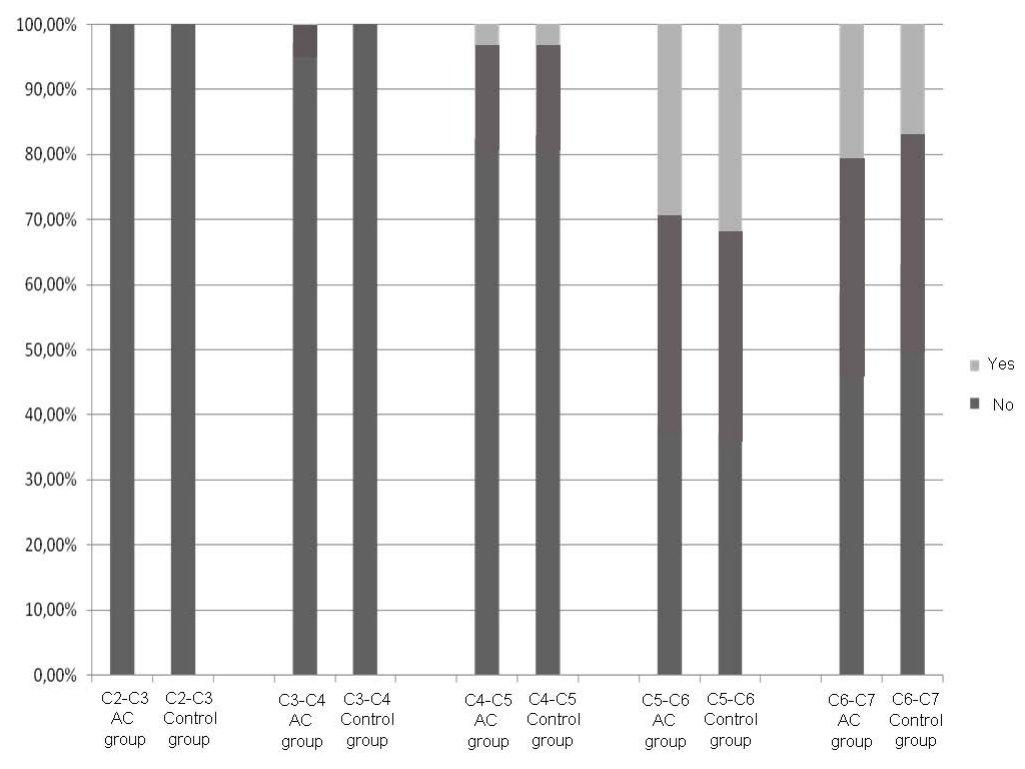
**Graphic representation of the prevalence of narrowing (< 4 mm) of the cervical intervertebral disc (p value from 0.63 to 0.98)**.

In the two studied groups, cervical osteophytes were observed mostly at the lower levels of C5-C6 and C6-C7; in almost 1/5 of the cases at C4-C5; rarely at C3-C4 and never at C2-C3 (Fig. 2). No statistical difference was registered between patients with chronic AC dislocation and subjects belonging to the control group (p value from 0.08 to 0.93). In the both groups, the narrowing of the intervertebral disc was seen frequently at the levels C5-C6 and C6-C7; very rarely at the level C4-C5 and never at C3-C4 and C2-C3 (Fig. 3). Once again, no statistical difference resulted comparing rates of the two groups (p value from 0.63 to 0.98). On the whole, 73.5% of the patients and 76% of the control subjects had at least one cervical level with degenerative changes (p = 0.23).

After dichotomisation, again, no significant association was found between the studied parameters and the mean lordosis value in the AC dislocation group (16°) and in the control group (26°) (p value ranging from 0.11 to 0.88)

**Figure 4:**
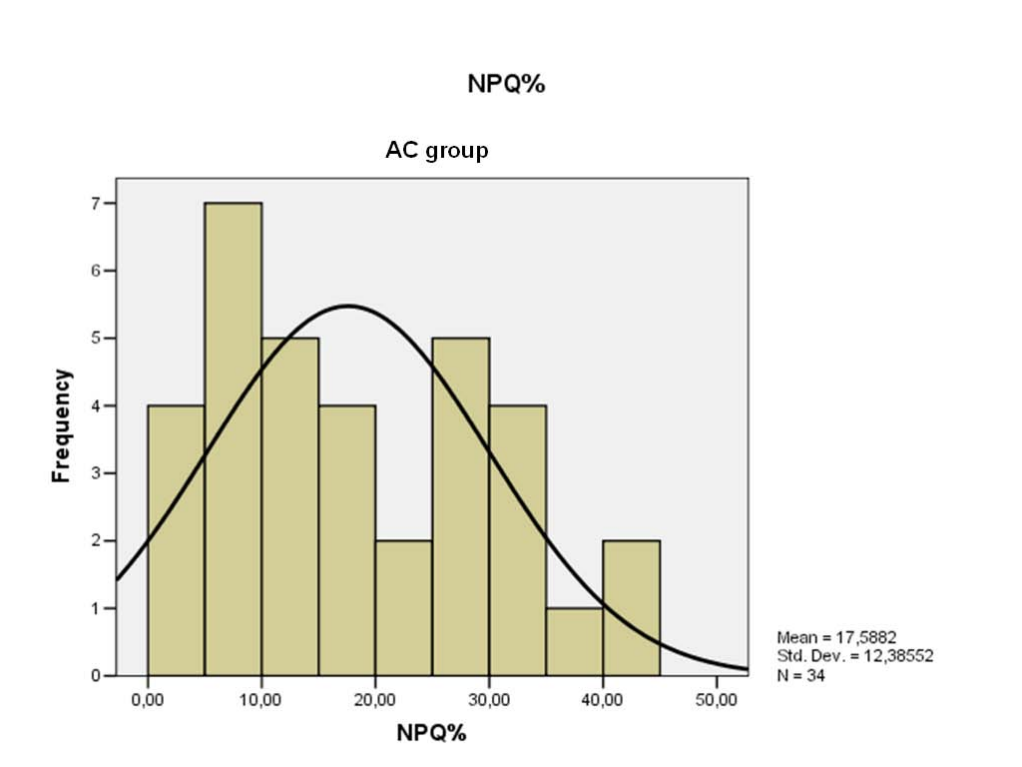
**NPQ results distribution obtained by the patients with chronic AC dislocation**.

**Figure 5:**
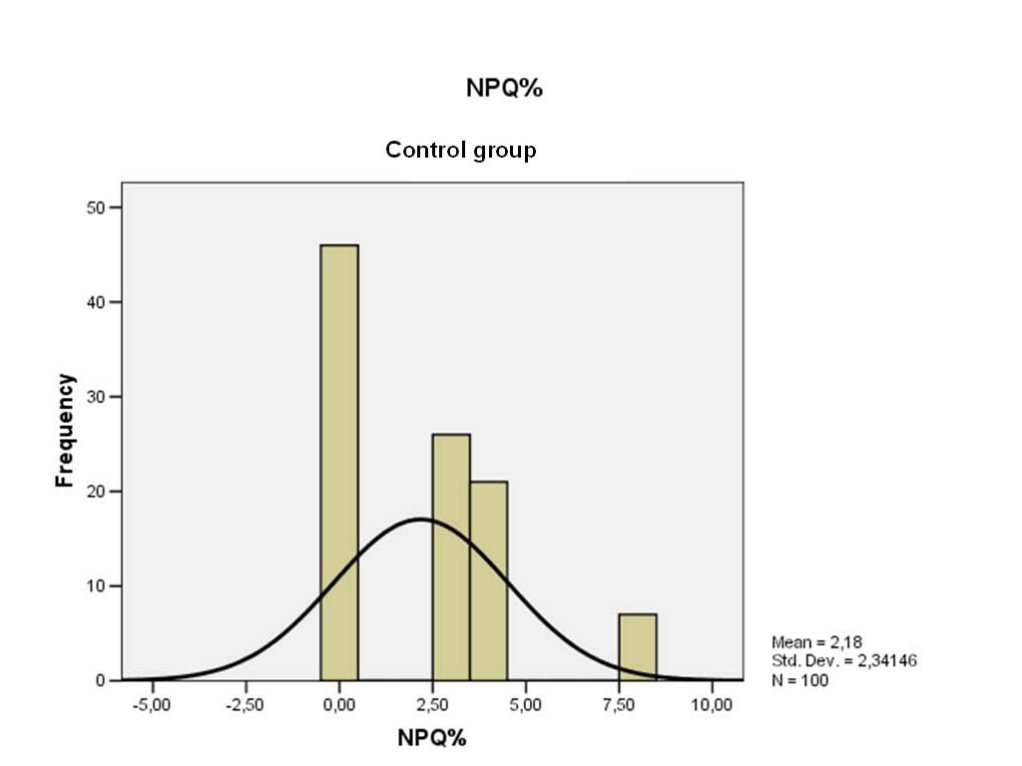
**NPQ results distribution obtained by the control group**. Note that the current scale is different with respect to the Fig. 4.

The NPQ mean value obtained by the patients with chronic AC dislocation was 17.58% (range: 0-44, S.D. 12.3) (Fig. 4); 2.18% (range: 0-8, S.D. 2.3) was the corresponding value obtained by the controls (Fig. 5) (p < 0.00001).

**Figure 6:**
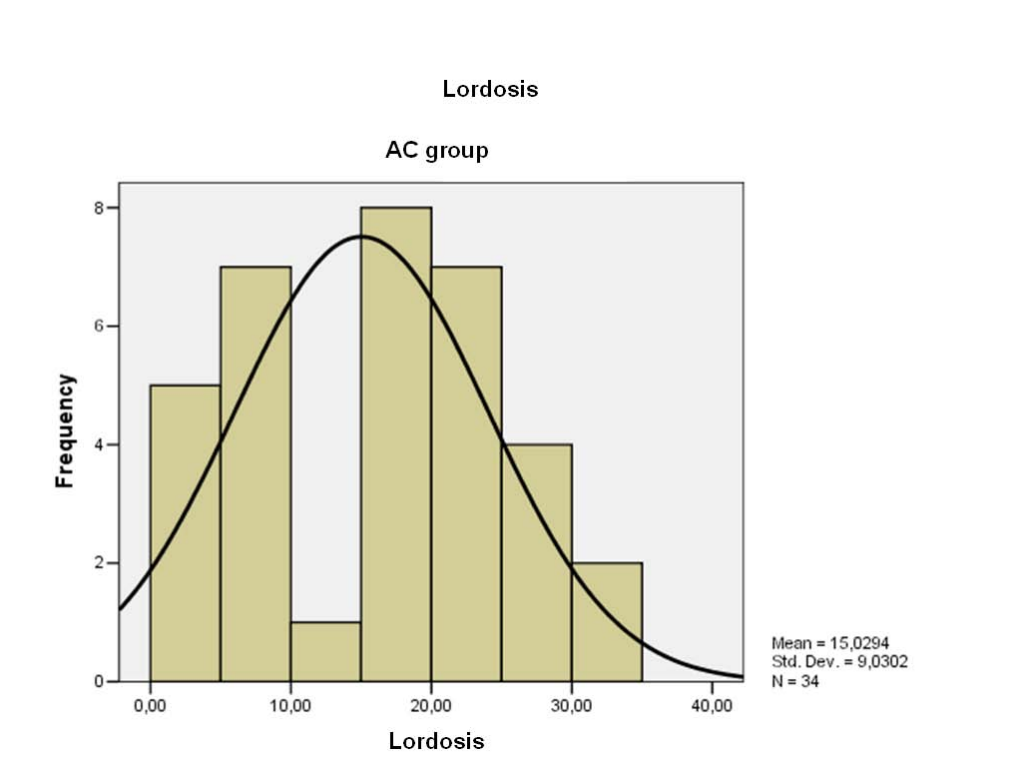
**Lordosis degree distribution obtained by the patients with chronic AC dislocation**.

**Figure 7:**
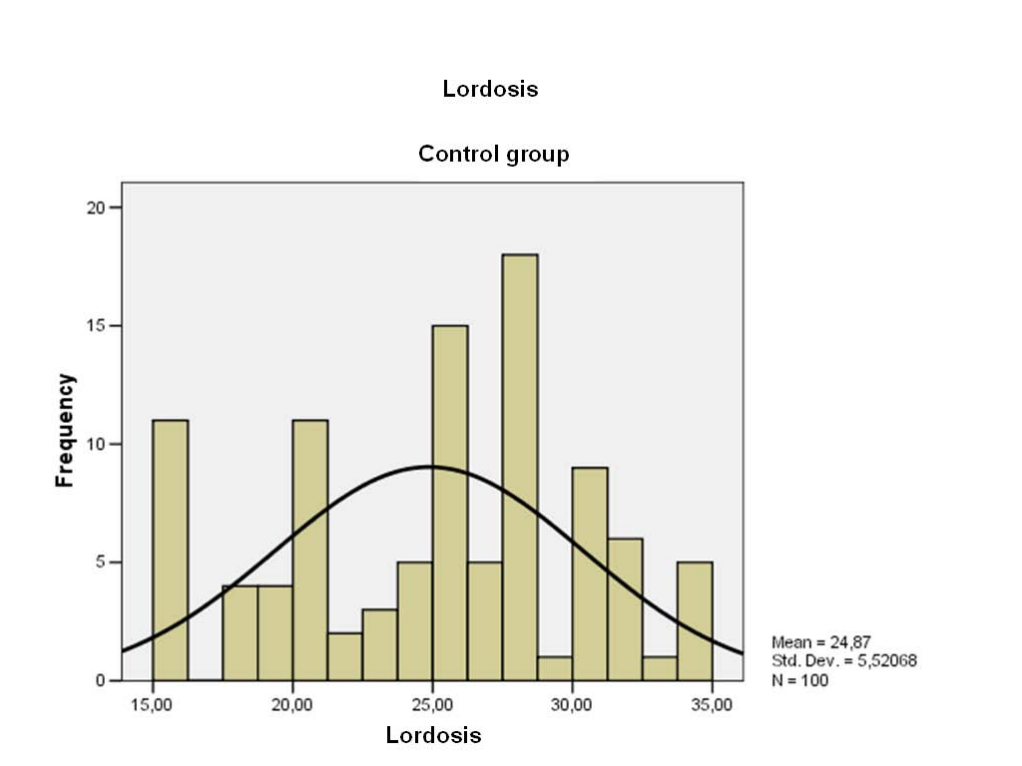
**Lordosis degree distribution obtained by the control group**. Note that the current scale is different with respect to the Fig. 6.

The average cervical lordosis was 15,02° (range: 3-33, S.D. 9.3) (Fig. 6) in patients with the AC joint dislocation and 24,87° (range: 15-35, S.D. 15.5) (Fig. 7) in the control group (p < 0.00001). Cervical lordosis was less than 27° in 76.4% of patients with chronic AC dislocation and in 52% of the control group (p < 0.001).

An inverse statistically significant nonparametric correlation was found between NPQ value and lordosis degree in the AC dislocation group (p = 0.001) while in the control group results are not correlated (p = 0.27).

The patients with chronic AC dislocation and the normal subjects with cervical lordosis more than 27° had a NPQ mean value of 7.5% and 2.8%, respectively (p = 0.053). The percentages arose, respectively, to 19.3% and 7.9% when cervical lordosis was equal or less than 27° (p = 0.041).

## Discussion

Patients with chronic Rockwood's type III AC dislocations can either be submitted to surgical stabilization or conservative treatment depending on whether or not the dislocation interferes with their daily activity, job, sports, shoulder strength and deformity. To our knowledge, this is the first paper recognising the fact that chronic AC dislocation may predispose cervical spine disorders. We hypothesized it as the trapezius muscle connects the clavicle, the scapula and the cervical spine with its fibres. We then assumed that an alteration of the tensioning of this muscle may cause cervical spine changes.

In the present series, both the osteophytosis and the narrowing of the intervertebral disc were commonly found at the levels C5-C6 and C6-C7 in the group of patients with chronic AC dislocation, while the upper cervical levels were only slightly, or not involved in degenerative changes. The same distribution in percentage was found in our control group and in the cohort of Hayashi et al. [10] who studied asymptomatic normal people aged 40 to 60. Since no statistical difference emerged when the patients with the asymptomatic subjects were compared, it is plausible as to whether or not the chronic AC dislocation interferes with osteophytes formation or the narrowing of the intervertebral disc. These changes, however, simply depend on aging symptoms. The limited role of degenerative changes on the genesis of the cervical spine pain has recently seemed to be confirmed by Yin and Bogduk [19], who observed that discogenic changes do not appear to be common among patients with chronic neck pain, and by Rao et al. [20], who stated that degenerative changes in the cervical spinal column are ubiquitous in the adult population, but infrequently symptomatic.

Cervical lordosis is generally measured using the Cobb's method [16]. This method has recently been criticized by Harrison et al. [17] for having a standard error of measurement; therefore, they proposed an alternative mathematical system. Although the latter may be considered a more accurate method, it is more complex and difficult to realize during the daily practice.

In recent literature, the role of hypolordosis as a cause of cervical pain has been an argument for discussion. Coté et al. [21] believe that controversies arise mainly from poor methodologic design and the use of measurement methods of unknown reliability in classifying degenerative changes and sagittal curve magnitude. McAviney et al. [22] observed however that only 4% of the patients with cervical kyphosis did not have cervicogenic symptoms. Gore et al. [23] instead, previously found an incidence of 9% of segmental kyphosis in 200 asymptomatic subjects, and found no subjects with a complete kyphosis from C2 trough C7. It is a common opinion that cervical hypolordosis may be the consequence of whiplash or other injuries such as muscular contractures or postural dysfunctions. In all cases, the causes of hypolordosis have been attributed to the dysfunction of the upper trapezius, cervical erector spinae, sternal head of the sternocleidomastoid and anterior scalene [24]. Chronic AC dislocation as cause of cervical disorders has never been reported. In our series, the average value of cervical lordosis, calculated with the Cobb's method, resulted statistically lower in patients with chronic AC dislocation than in the control group. Furthermore, three quarters of the patients, with respect to fifty percent of the controls, had a value lower than 27°, which is considered the average value in the asymptomatic general population [25-27].

Questions on the duration and intensity of the pain were good indicators in a patient's global assessment. The NPQ provided an objective measure to evaluate the outcomes in patients with acute or chronic neck pain. The analysis of the NPQ answers indicated that patients with chronic AC dislocation have more cervical troubles than the subjects belonging to the control group. We believe that cause of discomfort may be attributed in part to the AC separation, but above all to cervical hypolordosis. In fact, in both groups, those who were affected by hypolordosis had a higher NPQ average value than those who weren't. We don't know why chronic AC dislocation may be the cause of cervical hypolordosis; however, we believe that two possible explanations may be considered: a) the upper migration of the lateral end of the clavicle decreases the tension and length of the trapezius muscle, that is the only anatomical connector between clavicle and cervical spine; consequently, this condition may give repercussions to cervical spine; b) more simply, shoulder pain due to the AC dislocation may cause postural dysfunctions and thus cervical hypolordosis. Further EMG studies, performed on both sides, are needed for proving the first hypothesis; although a possible role of the upper trapezius on the genesis of the neck pain was already demonstrated [24]. It is however, also plausible that the two theories may coexist.

Beyond the lack of data regarding the EMG assessment of the trapezius, one limitation of our study is that we do not know the amount of cervical spine symptoms present before the injury. It therefore remains unclear as to whether the injury is truly responsible for the dysfunction, especially given the small number of patients studied. In addition, it may be possible that patients who had impaired cervical lordosis before their injury, degenerated after their injury. Finally, the average time elapsed from the dislocation is relatively small (average 38 months) to develop severe degenerative changes of the cervical spine.

Patients who sustained type III AC dislocation should be informed not only about repercussions that the AC separation causes on the involved shoulder, but also about the possibility that it may predispose to neck pain, and above all whether other risk factors already exist.

## Conclusions

In conclusion, our study shows that patients affected by chronic type III AC dislocation develop neck pain more frequently than the general population. We attributed the neck uneasiness to the cervical hypolordosis, in which prevalence is markedly higher than that observed in the control group.

The higher average NPQ values (100% as the worst result) were observed in patients with chronic AC dislocation, and mostly in those who developed cervical hypolordosis.

## Competing interests

The authors declare that they have no competing interests.

## Authors' contributions

SG evaluated the results of the study and participated in its design and coordination. He compared the radiographs; SC selected and recruited the study group; VA and AR showed the questionnaire to patient and to the control group; ARV performed the statistical analysis; FP helped to draft the manuscript. All authors read and approved the final manuscript.

## Pre-publication history

The pre-publication history for this paper can be accessed here:

http://www.biomedcentral.com/1471-2474/10/157/prepub
